# Dietary Polyphenols, Mediterranean Diet, Prediabetes, and Type 2 Diabetes: A Narrative Review of the Evidence

**DOI:** 10.1155/2017/6723931

**Published:** 2017-08-13

**Authors:** Marta Guasch-Ferré, Jordi Merino, Qi Sun, Montse Fitó, Jordi Salas-Salvadó

**Affiliations:** ^1^Department of Nutrition, Harvard T.H. Chan School of Public Health, Boston, MA, USA; ^2^Diabetes Unit, Center for Genomic Medicine, Massachusetts General Hospital, Boston, MA, USA; ^3^Channing Division of Network Medicine, Department of Medicine, Brigham and Women's Hospital and Harvard Medical School, Boston, MA, USA; ^4^Cardiovascular Risk and Nutrition (Regicor Study Group), Hospital del Mar Medical Research Institute (IMIM), Barcelona, Spain; ^5^CIBER de Fisiopatología de la Obesidad y Nutrición (CIBEROBN), Institute of Health Carlos III, Madrid, Spain; ^6^Human Nutrition Unit, University Hospital of Sant Joan de Reus, Department of Biochemistry and Biotechnology, Faculty of Medicine and Health Sciences, IISPV, Rovira I Virgili University, Reus, Spain

## Abstract

Dietary polyphenols come mainly from plant-based foods including fruits, vegetables, whole grains, coffee, tea, and nuts. Polyphenols may influence glycemia and type 2 diabetes (T2D) through different mechanisms, such as promoting the uptake of glucose in tissues, and therefore improving insulin sensitivity. This review aims to summarize the evidence from clinical trials and observational prospective studies linking dietary polyphenols to prediabetes and T2D, with a focus on polyphenol-rich foods characteristic of the Mediterranean diet. We aimed to describe the metabolic biomarkers related to polyphenol intake and genotype-polyphenol interactions modulating the effects on T2D. Intakes of polyphenols, especially flavan-3-ols, and their food sources have demonstrated beneficial effects on insulin resistance and other cardiometabolic risk factors. Several prospective studies have shown inverse associations between polyphenol intake and T2D. The Mediterranean diet and its key components, olive oil, nuts, and red wine, have been inversely associated with insulin resistance and T2D. To some extent, these associations may be attributed to the high amount of polyphenols and bioactive compounds in typical foods conforming this traditional dietary pattern. Few studies have suggested that genetic predisposition can modulate the relationship between polyphenols and T2D risk. In conclusion, the intake of polyphenols may be beneficial for both insulin resistance and T2D risk.

## 1. Introduction

In the past few decades, type 2 diabetes (T2D), in parallel with the obesity epidemic, has become a public health challenge for many countries [1]. Increasing evidence has demonstrated that the combinations of several unhealthy lifestyle factors, including a sedentary lifestyle, unhealthy diets, overweight/obesity, smoking, and excessive alcohol intake, were responsible for 90% of T2D cases [2]. For this reason, strategies focused on lifestyle and the promotion of a healthy diet to prevent T2D have been identified as a cornerstone of researchers and policymakers.

Recently, growing interest has emerged on the beneficial effects of plant-based diets for the prevention of chronic diseases including obesity, diabetes, and cardiovascular disease [3, 4]. Such diets are based on foods derived from plants, including fruits, vegetables, whole grain cereals, legumes, and nuts, with limited animal products. As an example, the Mediterranean diet, which has been associated with many health benefits [5], is characterized by a high intake of fruits, vegetables, legumes, nuts, and olive oil; a moderate consumption of dairy products and wine; and low intake of red and processed meat, butter, cream, and sugar drinks. One of the dietary constituents common in plant-based diets are polyphenols, which are especially abundant not only in fruits, vegetables, whole grains, and legumes but also in cocoa, tea, coffee, and red wine [6].

Polyphenols are a large and heterogeneous group of phytochemicals containing phenol rings and are divided into flavonoids, phenolic acids, stilbenes, and lignans [7] (Table 1). Flavonoids are classified into flavones, flavonols, flavanols, flavanones, isoflavones, and anthocyanins [8]. Among others, fruits like apples, grapes, pears, and berries typically contain high amounts of polyphenols (200–300 mg per 100 g) [9]. The most common phenolic acids are caffeic acid and ferulic acid, which is a major phenolic compound in coffee and cereals, respectively [10]. The best-studied stilbene is resveratrol in grapes, grape products, and red wine [10]. Other main dietary sources of polyphenols include vegetables, chocolate, tea, whole grains, dry legumes, nuts, and olive oil [10]. Polyphenols are the most abundant antioxidants in the diet, and their intake has been associated with a reduced incidence of T2D in humans [11–13]. Polyphenols have anti-inflammatory effects and may influence glycemia through different mechanisms, including the inhibition of glucose absorption in the gut and the improvement of insulin resistance [9].

This review aims to summarize the relevant evidence linking dietary polyphenols to prediabetes and T2D with a focus on polyphenol-rich food characteristics of the Mediterranean diet (MedDiet). In addition, the present work aims to describe genotype-polyphenol interactions and metabolic biomarkers to understand the relationship between polyphenols and T2D.

## 2. Dietary Polyphenols, Insulin Sensitivity, and Resistance

Abundant evidence generated in human studies collectively suggests that the intake of polyphenols and their major food sources may exert beneficial effects on improving insulin resistance and related diabetes risk factors, such as inflammation and oxidative stress (as reviewed by Scalbert et al. [9]). Given the amount of such evidence, this narrative review does not seek to provide a comprehensive summary of all study findings, but rather focuses on well-conducted clinical trials and observational prospective studies. In this regard, only randomized and controlled trials have been considered, and regarding the prospective cohort studies, only those with appropriate control of potentially confounding variables have been included. We have performed a narrative review in MEDLINE up to June 2017. Our search terms combined the exposures (polyphenols, flavonoids, phenolic acids, stilbenes, lignans; and also, MedDiet, extra-virgin olive oil, nuts, and red wine) with several outcomes (insulin resistance, insulin sensitivity, oxidation, inflammation, and T2D). We also conducted a specific search for well-powered human clinical studies with the potential for clinical translational findings. We reviewed the evidence of polyphenols and T2D from a nutritional genomic perspective, particularly from metabolomics and metagenomics. We acknowledge other omics studies using transcriptomics, proteomics, and epigenomics may also have an important role on understanding polyphenol mechanisms of action, but given the clinical translational nature of this review, we have not included these studies.

Among all polyphenols, the beneficial effects of flavanols (flavan-3-ols) and their primary food sources, including cocoa, chocolate, and red wine, have been most widely examined in clinical trials. In a meta-analysis of 24 trials among 1106 individuals that examined the effects of cocoa intake for 2–18 weeks, Shrime et al. showed significant effects of cocoa intake on improving insulin sensitivity [14]. Homeostatic model assessment of insulin resistance (HOMA-IR) decreased by 0.94 points (95% CI = 0.59, 1.29; *P* < 0.001) with the consumption of flavonoid-rich cocoa. This meta-analysis included healthy participants and patients with hypertension, diabetes, and overweight. Total flavonoid intake ranged from 16.6 mg/d to 1080 mg/d and control group included low-flavonoid cocoa, white chocolate, skim milk, and placebo capsules. In nine studies, at least one author was employed by a chocolate company [14]. In another meta-analysis of 42 trials comprised of 1297 individuals which was conducted in 2012, Hooper et al. showed that acute to short-term (≤18 weeks) intake of cocoa, chocolate, and flavan-3-ols significantly decreased insulin resistance [15]. Healthy participants and participants with elevated blood pressure, serum cholesterol, and diabetes as well as other health conditions were included. Interventions were cocoa drinks, dark or milk chocolate, cocoa supplements, solid chocolate plus cocoa drinks, and a whole diet (all foods provided) including cocoa powder and chocolate. These were compared with low flavan-3-ol versions of the same foods, drinks, or supplements [15].

Data on the effects of long-term chronic consumption of flavan-3-ols on cardiometabolic health are scarce. In a randomized, placebo-controlled trial in 93 patients with T2D, the intake of 7 grams per day of flavonoid-enriched chocolate (containing 850 mg flavan-3-ols and 100 mg isoflavones) for 1 year significantly improved insulin resistance. In this long-term trial, effects on hemoglobin A1c (HbA1c) and glucose were not observed [16]. However, the authors acknowledged that the dropout rate was high and limited to postmenopausal women receiving diabetes therapy.

Another food source of flavan-3-ols, green tea, has also been extensively examined in short-term clinical trials evaluating its beneficial effects on cardiometabolic health. For example, catechins from green tea exerted beneficial effects on lowering fasting glucose, although effects on fasting insulin, HbA1c, and HOMA-IR were not significant in a meta-analysis summarizing results of 22 randomized clinical trials with 1584 participants and ranging interventions from 3 to 24 weeks [17]. Green tea catechin dosage ranged from 240 mg/d to 1207 mg/d and was compared with water or placebo.

Clinical trials that aim to assess the effects of anthocyanins are relatively sparse. In a double-blinded 6-week clinical trial among 32 obese nondiabetic individuals, Stull et al. found that intake of a smoothie with added blueberry powder (equivalent to 668 mg/day anthocyanins) as compared to a smoothie of equal nutritional value without added blueberry bioactivities significantly improved insulin sensitivity measured by a hyperinsulinemic-euglycemic clamp [18]. Beneficial effects of anthocyanins (either purified anthocyanin (320  mg/d) derived from bilberry and black currant or placebo) on improving insulin resistance were also observed in a 12-week trial conducted in 74 patients with nonalcoholic fatty liver disease [19]. Of note, the descriptions of the control foods and extracts used as in the abovementioned clinical trials were poorly described and no indication on its nature is given.

The consumption of beverages with added freeze-dried strawberries rich in anthocyanins compared to placebo for 12 weeks did not exert effects on inflammatory markers or glucose measurements in 60 individuals with abdominal adiposity and hypercholesterolemia, although a significant reduction of total and low-density lipoprotein (LDL) cholesterol was observed in this trial [20].

Isoflavones are a class of flavonoids with estrogenic effects that are found in soya beans and are highly consumed in East Asian populations. Probably because of these estrogenic properties, most of the clinical trials evaluating the effects of isoflavone supplementation focused on postmenopausal or perimenopausal women. In a meta-analysis of 24 trials comprised of 1518 men and women, intervention groups included isoflavone extracts as supplements and whole soy foods into the diet and compared the isolated soy protein that contained isoflavones with milk or animal protein. The soy protein content ranged from 0 to 40 g soy protein/d, and the isoflavone content ranged from 36 to 132 mg isoflavones/d. Results from this meta-analysis have shown that soy intake did not exert significant effects on measures of glucose metabolism, including fasting glucose and insulin, HOMA-IR, HbA1c, and 2-hour glucose or insulin levels [21]. Restricting the analysis to postmenopausal women generated similar results.

Interestingly, in another meta-analysis that only considered clinical trials conducted among non-Asian perimenopausal or postmenopausal women, soy isoflavones or genistein supplements for 3 months to 2 years significantly reduced serum insulin and HOMA-IR, but had no effects on fasting blood glucose [22].

Lignans are a group of polyphenols that also possess estrogenic properties. A few trials have been conducted to elucidate the effects of flaxseed supplements that contain high amount of lignans on insulin resistance and other cardiometabolic intermediate outcomes. In a small crossover trial among 9 obese subjects with glucose intolerance, flaxseed intake (40 g/day) compared to 40 g wheat bran for 12 weeks significantly improved insulin resistance [23]. In another small randomized trial comparing flaxseed intake (40 g/day) with hormone replacement therapy among 25 postmenopausal women, flaxseed intake significantly reduced glucose and insulin levels, although these benefits were comparable to those of hormone replacement therapy [24]. However, sample sizes of these studies were small and replication of the results in other populations is needed to make strong conclusions. In a crossover, randomized trial conducted among ~70 patients with T2D, Pan et al. showed that supplementation of 360 mg/day of lignans derived from flaxseed for 12 weeks significantly reduced HbA1c [25] and C-reactive protein (CRP) [26] in comparison with the placebo group. Interestingly, such effects were primarily observed in women, whilst no effects on CRP levels were found in men [26]. In the largest trial testing the efficacy of flaxseed supplements so far, flaxseed lignan supplements (543 mg/day) (as compared to placebo) did not exert significant effects on lowering fasting blood glucose concentrations or inflammatory markers in 100 Canadian healthy men and women followed for 6 months [27]. Moreover, in a recent trial among 99 prediabetic patients, flaxseed supplementation did not improve insulin sensitivity [28]. This randomized, clinical trial compared two groups receiving 40 g and 20 g per day of flaxseed powder daily for 12 weeks and the third group was the control group (no intervention) [28].

Evidence regarding other polyphenols or their food sources is sporadic. In a crossover trial conducted in overweight men and women, supplementation of 150 mg/day resveratrol compared to placebo capsules for 4 weeks had no effects on insulin sensitivity or inflammatory markers [29]. Supplements of chlorogenic acid (1 g/d, a polyphenol highly present in coffee, significantly reduced glucose and insulin concentrations 15 minutes following an oral glucose tolerance test (OGTT), although no overall improvement on OGTT measurements was observed in a small crossover trial (three interventions: 12 g decaffeinated coffee, 1 g chlorogenic acid, 500 mg trigonelline, and placebo (1 g mannitol)) including 15 overweight men [30]. Of note, trials that examined the effects of coffee consumption per se did not generate consistent findings regarding whether insulin resistance could be improved, although all these trials were small in sample size (ranging from 10 to 47 participants) [31–33]. Of note, because the portions of coffee are larger in North European and American populations and therefore the intake of polyphenols from this food greater, some differences may exist among populations.

In summary, intake of polyphenols, especially flavan-3-ols and their food sources, have demonstrated overall beneficial effects on decreasing insulin resistance, chronic systematic inflammation, oxidative stress, and improving other cardiometabolic risk factors. However, some publication bias may be present as several clinical trials have been sponsored by the food industry, particularly for cocoa, chocolate, and tea, and this may explain the greater amount of evidence on this food groups. In addition, some clinical trials on this topic were of short duration which may dilute the effect of polyphenol intake on a long term. Although surrogate endpoints measured in most clinical trials can help to monitor changes during follow-up and are relevant for the prediction of T2D, more research is warranted to elucidate the metabolic effects of polyphenols in larger trials with longer intervention term and risk of T2D. In this regard, the ongoing Cocoa Supplement and Multivitamin Outcomes Study (COSMOS) [34], which aims to evaluate the efficacy of a concentrated cocoa extract using a five-year randomized trial among 18,000 healthy men and women, may provide conclusive evidence on the health benefits of cocoa (rich in polyphenols) on cardiometabolic hard outcomes. Finally, novel metabolomic techniques will help to determine more objective plasma concentrations of polyphenols and had a better picture of availability and potential beneficial effects on insulin resistance and T2D.

## 3. Dietary Polyphenols and Risk of Type 2 Diabetes

Table 2 summarizes the evidence on prospective studies evaluating polyphenol intake and risk of T2D. Several studies have evaluated the associations between the intake of total flavonoids or specific flavonoids and the risk of T2D. Four studies including data from 6 cohorts on healthy participants have evaluated the associations between dietary total flavonoids and risk of T2D [35]. Results from a meta-analysis including 18,146 incident T2D cases and 284,806 participants showed that the relative risk of T2D for the highest intake of total flavonoids compared with the lowest was 0.91 (95% CI: 0.87, 0.96). A dose-response analysis of these results revealed that 500 mg/d increase in total flavonoid intake was associated with a significant 5% reduction of T2D incidence. These beneficial effects were especially observed in younger individuals and in those studies with larger follow-up [35]. Two new studies not included in this meta-analysis were published later [36, 37]. In the European Prospective Investigation into Cancer and Nutrition-InterAct (EPIC-InterAct) study, an inverse association between total flavonoid intake and T2D risk was reported in a case-cohort study including 12,403 incident cases of diabetes and 16,154 healthy participants [36]. In contrast, total flavonoid intake was nonsignificantly associated with the risk of T2D in a report from the Framingham Offspring Study, which included 2915 participants followed for 11.9 years [37].

Several studies have evaluated the association between the consumption of different subtypes of flavonoids and T2D risk with some controversial results. The Finnish Mobile Clinic Health Examination Survey (FMCHES) [38], the Womens' Health Study [39], both including healthy participants, and the *α*-Tocopherol, *β*-Carotene Cancer Prevention (ATBC) Study, which included male smokers [40], examined the associations between the intake of total or selected flavonol or flavone molecules (quercetin, kaempferol, myricetin, hesperetin, and naringenin) and T2D risk. In the FMCHES, higher intake of quercetin and myricetin showed a trend towards a reduction in the risk of T2D, but not for the rest of the compounds [38]. No significant associations were observed between total or selected types of flavonol and flavone intake and T2D in the Women's Health Study [39] and the ATBC Study cohorts [40].

Findings from large-scale prospective studies have evaluated the associations between flavonoids and T2D risk. After pooling the results of three large cohorts (Nurses' Health Study (NHS), NHSII and Health Professionals Follow-up Study (HPFS); 3,645,585 person-years of follow-up; 12,611 incident cases of diabetes), higher intakes of anthocyanins and anthocyanin-rich fruits were associated with a decreased risk of T2D after adjusting for multiple potential confounders, whereas no associations were shown for total flavonols, flavones, flavanones, and flavan-3-ols [11]. A case-cohort study within the frame of the EPIC-InterAct cohort, which included healthy middle-aged participants from eight countries, showed that when comparing extreme quintiles of consumption of flavonoid subclasses, total flavonols and flavanols, including flavan-3-ol monomers, were inversely related to the diabetes risk, whereas no associations were reported for the intake of lignans [36]. It is important to highlight that these associations were observed within the context of a European-wide population with a large heterogeneity in the intake of these compounds. In a following report from the same study [41], significant inverse trends for the intakes of proanthocyanidin dimers and trimers and incident T2D were observed, but not for proanthocyanidins with a greater polymerization degree. Among the flavonol subclasses, only myricetin was associated with a lower incidence of T2D [41]. Finally, in the Framingham Offspring Cohort, each 2.5-fold increase in flavan-3-ol intake was marginally associated with an 11% lower incidence of T2D, but no other associations between other flavonoid classes (flavonols, flavones, flavanones, anthocyanins, and polymeric flavonoids) and risk of T2D were reported [37].

In relation to the intake of isoflavones, findings from the NHS, NHSII, and HPFS have shown inverse associations for total isoflavones and major individual isoflavones, especially those present in soy and soy products, including daidzein and genistein, with the risk of developing diabetes [42]. However, no significant association between total isoflavone intake and T2D has been reported in the EPIC-InterAct Study [36].

In summary, results from prospective cohort studies have suggested inverse associations between the intake of total flavonoids and specific flavonoid subclasses and the risk of T2D although some controversial results exist.

## 4. Characteristic Polyphenols of the Mediterranean Diet, Prediabetes, and Type 2 Diabetes

As described before, dietary polyphenols come mainly from plant-based foods [8]. Olive oil, nuts, red wine, legumes, fruits, and vegetables, key components of the MedDiet, are all polyphenol-rich foods [5]. The much-appreciated MedDiet is well-known for its antioxidant and anti-inflammatory effects [43, 44]. The cardioprotective effect of this dietary pattern has been attributed, in part, to the high amount of antioxidant components such as the phenolic compounds [43]. Moreover, the MedDiet has been shown to be beneficial for glycemic control and T2D [45, 46]; however, few studies have evaluated the effects of specific polyphenols from food products characteristic of this diet and insulin resistance or T2D. In this section, we aim to provide further information on characteristic food of the MedDiet that contain large amounts of polyphenols.

### 4.1. Extra-Virgin Olive Oil

Extra-virgin olive oil is probably one of the components that most differentiates the MedDiet from other dietary patterns. Phenolic components such as oleuropein and hydroxytyrosol, flavonoids, specially flavones, and lignans are abundant in extra-virgin olive oil [47]. Extra-virgin olive oil is the best quality olive oil, is rich in taste and color, and also contains high amount of bioactive compounds compared to other types of olive oil (such as common olive oil, which is refined, has less flavor, color, and aroma, and contains fewer amounts of antioxidants and vitamin E). The phenolic composition of olive oil ranges from 50 to 800 mg/L depending on several factors like the variety, cultivation techniques, degree of ripeness, and climate, among others [48].

Evidence from clinical trials on the effects of olive oil polyphenols and biomarkers of T2D is scarce. Only two randomized trials have been published so far evaluating the effect of olive leaf polyphenols on markers of insulin sensitivity [49, 50]. Main polyphenols in olive leaves (oleuropein and hydroxytyrosol) are similar than those in fruit and fruit oil; however, its concentration is greater. In the first intervention study, including 70 adults with T2D, those participants consuming olive leaf extract (500 mg) exhibited significantly lower HbA1c and fasting plasma insulin levels; however, postprandial plasma insulin levels did not differ significantly by treatment group [50]. The second study demonstrated that participants who received capsules of olive leaf extract for 12 weeks (51.1 mg oleuropein and 9.7 mg hydroxytyrosol per day) compared to placebo improved pancreatic *β*-cell function by 28% and also significantly improved insulin sensitivity compared to the placebo [49]. Other clinical trials have evaluated the effect of extra-virgin olive oil rich in polyphenols on glycemic biomarkers [51, 52]. Daily consumption of polyphenol-rich extra-virgin olive oil for 8 weeks significantly reduced fasting plasma glucose and HbA1c, as well as other circulating inflammatory adipokines, in overweight patients with T2D [52]. In contrast, no significant effects on fasting glucose after supplementation with 20 mL per day of polyphenol-rich olive oil during 6 weeks compared to 20 mL dose of low phenolic oil were observed in healthy participants [51]. The PREDIMED trial has provided conclusive evidence of the beneficial effects of a MedDiet supplemented with extra-virgin olive oil on glucose metabolism and T2D [45, 46]. In this regard, a MedDiet supplemented with extra-virgin olive oil reduced the risk of T2D by 40% (HR: 0.60; 95% CI: 0.43, 0.85) after a median of 4.1 years of follow-up among participants at high cardiovascular risk compared to the control group [46]. Previous reports from the PREDIMED Study also showed that a MedDiet supplemented with extra-virgin olive oil reduced fasting plasma glucose improved insulin resistance and inflammatory biomarkers [45, 53]. Another randomized crossover trial in healthy volunteers, the EUROLIVE study, which tested the effect of the daily 3-week administration of 25 mL of 3 olive oils with low, medium, and high phenolic content, demonstrated beneficial effects of phenolic compounds on oxidative damage on lipids and HDL cholesterol [54]. However, given that the volunteers were healthy, no effects were observed on glucose levels; nevertheless, the objective of the study was the lipid and lipoperoxidation. Also in a postprandial state study with healthy volunteers, after 50 mL of virgin olive oil load, the expression of candidate genes related to insulin sensitivity was observed in peripheral mononuclear cells [55].

### 4.2. Nuts

Nuts, which are well-known for their unique nutritional composition (rich in unsaturated fatty acids, fiber, antioxidant vitamins, minerals, and other bioactive compounds), are also consumed in high amounts in the MedDiet. Several seeds and nuts are among the richest sources of polyphenols: chestnuts and walnuts are rich in ellagitannins; while hazelnuts, pecan, and almonds are rich in proanthocyanidins; and flaxseed is rich in lignans [8]. Although the effects of nut consumption on glycemic markers and T2D have been well studied over the past decades [56, 57], specific studies on polyphenols from nuts are lacking. However, we have some indirect evidence that polyphenols from tree nuts may have beneficial effects on glucose metabolism. Some evidence has suggested that ellagic acid, which is found in a considerable amount in several nuts (especially walnuts), could be beneficial for diabetes control [58]. In addition, urolithin A glucuronide in plasma, a metabolite of dietary ellagic acid derivatives which has shown to be a discriminative biomarker of nut exposure, was inversely correlated with insulin resistance measured by HOMA-IR. However, further clinical trials are needed to confirm these associations [59].

### 4.3. Red Wine

One of the main characteristics of the traditional MedDiet is the moderate intake of wine consumed with meals. Wine, particularly red wine, is rich in phenolic compounds including flavonoids (anthocyanins, tannins, and catechin), stilbenes like resveratrols, tyrosols, and hydroxytyrosols. Evidence suggests that red wine consumption exerts some benefits on cardiovascular health [60]; however, whether these effects are due to ethanol or to nonalcoholic components of red wine is still unclear. Along these lines, a randomized clinical trial including sixty-seven men at high cardiovascular risk was designed to compare the effects of moderate intake during 4 weeks of red wine, a high polyphenolic alcoholic beverage (the equivalent amount of dealcoholized wine), a high polyphenolic nonalcoholic beverage, and gin (without polyphenols) on glucose metabolism and lipid profile [61]. Findings of that study suggested that red wine rich in polyphenols with or without alcohol, but not gin, improved glucose metabolism, as measured by HOMA-IR [61]. Previous clinical trials have demonstrated improvements in insulin sensitivity with the consumption of red wine [62] and red grape juice [63], but others reported no significant effects on these parameters [64, 65]. In addition, the positive effects on glucose metabolism of supplementation with grape derived polyphenol and resveratrol, one of the main polyphenols in red wine, have been observed in intervention studies [66, 67]. Finally, a 2-year randomized clinical trial in patients with T2D suggests that initiating moderate red wine intake, among well-controlled diabetics as part of a healthy diet, is apparently safe and modestly decreases cardiometabolic risk [68]. Moreover, genetic interactions may play a role in glucose metabolism, and red wine's effects also involve nonalcoholic constituents such as polyphenols. In this trial, slow ethanol metabolizers (alcohol dehydrogenase alleles [ADH1B∗1] carriers) significantly benefited from the effect of red wine on glycemic control (fasting plasma glucose, HOMA-IR, and hemoglobin A1c) compared with fast ethanol metabolizers (persons homozygous for ADH1B∗2) [68].

### 4.4. Other

Fruits, vegetables, whole grains, legumes, and coffee are also primary sources of polyphenols [8] and are beneficial for glycemic control and prevention of T2D [4, 69]. Other beverages and foods that are rich in polyphenols include green tea, soy, chocolate, and cocoa and could also play a role in the prevention of T2D [70].

In summary, the MedDiet and its key components, extra virgin olive oil, nuts, and red wine, are inversely associated with insulin resistance and T2D. To some extent, these effects may be attributed to its high amount of polyphenols and other bioactive compounds.

## 5. New Frontiers in Nutrition Research: Genotype-Polyphenol Interaction on Type 2 Diabetes

**Figure 1:**
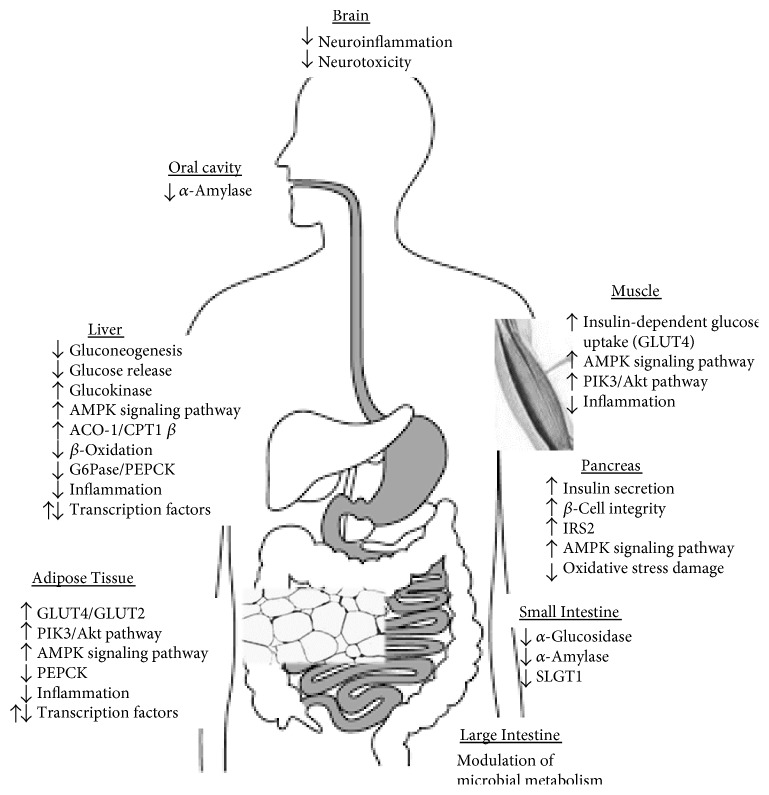
Relevant mechanisms linking dietary polyphenols and T2D risk. Polyphenols can exert a beneficial effect on type 2 diabetes by a number of mechanisms including (a) slowing carbohydrate digestion and glucose absorption by interacting with oral cavity and intestinal *α*-amylase and intestinal *α*-glucosidase and sodium-dependent glucose transporter (SLGT1); (b) stimulating insulin secretion in the pancreas via increasing 5′ adenosine monophosphate-activated protein kinase (AMPK) pathway, and insulin receptor substrate (ISRS) and decreasing *β*-cell oxidative damage which preserves *β*-cell integrity; (c) modulating liver glucose release due to increase in acyl-CoA oxidase 1 (ACO-1) and carnitine palmitoyl transferase 1-*β* (CPT1-*β*) and diminishing glucose 6 phosphatase (G6Pase) and phosphoenolpyruvate carboxylase (PEPCK); and (d) activation of glucose uptake receptors in the insulin-sensitive tissue. Additionally, the modulation of microbial metabolism can synergically benefit glucose homeostasis.

Because of their chemical structures, dietary polyphenols exert multiple activities by interacting with several molecular pathways particularly relevant for glucose homeostasis. In Figure 1, we summarize relevant mechanisms linking dietary polyphenols and T2D risk. These include slowing carbohydrate digestion and glucose absorption, stimulation of insulin secretion, modulation of glucose release, and activation of insulin receptors and glucose uptake in the insulin-sensitive tissues [71]. A growing body of evidence from in vitro and animal studies has shown that polyphenols can activate and/or silence transcription factors, and consequently influence gene expression, and regulate different signaling pathways in the muscle, the liver, pancreatic *β*-cells, the hypothalamus, and adipose tissue, thereby contributing to glucose homoeostasis [72, 73].

Although promising data aimed at understanding how different polyphenol classes can modulate genetic regulation and expression have been published, few studies have investigated whether genetic predisposition modifies the relationship among polyphenols, intermediate phenotypes of insulin resistance, and T2D risk [74–79]. An initial and very preliminary track of evidence for genotype-polyphenol interaction is emerging from studies of coffee, the consumption of which is highly spread in Mediterranean regions. In a prospective population-based cohort study including 4077 normal glycemic individuals over a 4-year follow-up, habitual coffee intake outweighed the hazard of unfavorable genetic predisposition on 3 well-known T2D-increasing risk genetic loci, including *IGF2BP2*, *CDKAL1*, and *KCNJ11* [74]. Along these lines, an independent study including 1180 nondiabetic young to middle-aged participants with stage 1 hypertension, baseline coffee consumption was longitudinally associated with the risk of impaired glucose tolerance only in carriers of CYP1A2∗1F allele. Among participants homozygous for the ∗1A allele, which is responsible for fast caffeine metabolism, the favorable action of polyphenols or other bioactive agents balanced the genetic and metabolic risk for T2D [75]. Finally, in a prospective epidemiological study from the EPIC-InterAct cohort, including 8086 incident T2D cases in 11,035 participants over 12.5 years, habitual coffee consumption was associated with a 7% T2D risk reduction among carriers of the diabetes increasing risk allele at transcription factor 7-like 2 locus (TCF7L2) [76]. In addition, an interaction between an incretin-specific genetic risk score, designed to capture the genetic predisposition to defects in postprandial insulin secretion and coffee consumption on T2D risk was observed (i.e., each additional cup of coffee was associated with 5% lower T2D risk in individuals carrying high number of risk alleles). In line with previous results, adherence to the MedDiet has been reported to be able to reduce the adverse effect of the TCF7L2 polymorphism on fasting glucose and blood lipids and, importantly, on stroke incidence [77]. Unfortunately, with this type of study, we cannot differentiate the effect of polyphenols from other dietary compounds of the MedDiet.

Despite nutrigenomic evidence being still preliminary, and major challenges existing in analytical strategies and replication, the role of polyphenol-rich components on TCF7L2 locus is likely to be relevant. TCF7L2 is the strongest common genomic region associated with T2D (OR = 1.4) and encodes a transcription factor for proteins involved in the proper functioning of the Wnt signaling pathway, essential for insulin secretion and beta-cell proliferation [78, 79]. It would be rational, therefore, to envision that nutrients which tend to stimulate both insulin and incretin secretion will tend to partly compensate for any genetic defects in the incretin system and thus confer higher protection from T2D in individuals carrying the predisposing polymorphisms. Understanding gene-diet interactions in T2D holds promise to achieve the goal to develop personalized diet and lifestyle interventions based on genetic profile; nevertheless, further research is warranted before implementing preliminary results to clinical practice is still required.

## 6. Biomarkers of Polyphenol Intake

Along with the development of sophisticated genomics techniques, recent advancements in analytic procedures have allowed the measurement of molecules that provide a close representation of what is encoded by the genome and modified by diet, gut microbiome, and other environmental exposures [80]. In this regard, metabolomics has greatly contributed in the identification of new biomarkers (of dietary exposure, nutritional status, and health/disease). In a prospective nested case-control study including 1107 diabetic women and 1107 control women participating in the NHS, metabolites from different classes of polyphenols were associated with T2D risk. For example, urinary excretion of enterolignans, especially enterolactone, was significantly associated with a lower T2D risk during up to 13 years of follow-up [12]. Comparing the extreme quartiles of urinary excretion of enterolactone, the relative risk of T2D was 0.62 (95% CI: 0.44, 0.88; *P* for trend = 0.003). In contrast, metabolites from flavanones (naringenin and hesperetin), flavonols (quercetin and isorhamnetin), and phenolic acids (caffeic acid) in spot urine samples were significantly associated with a lower T2D risk within a relatively short period of follow-up (≤4.6 years) after sample collection, but not during the entire follow-up [81]. Such a temporal pattern may be explained by the significant variability of these metabolites over time, especially when they are measured in spot urine samples [81]. In a separate analysis, Ding et al. found an inverse association between urinary excretion of daidzein and genistein, but not other isoflavone metabolites, and T2D risk in the NHS and NHSII [82]. An inverse association between isoflavone biomarkers and diabetes risk was also observed in two other prospective analyses conducted among minority populations. Among 431 Native American participating in the Strong Heart Family Study [83], Zhao et al. used an untargeted metabolomics approach to examine metabolite levels in plasma. From seven metabolites related to diabetes risk after adjustment by multiple testing, an isoflavone metabolite [(3S)-7-hydroxy-2′,3′,4′,5′,8-pentamethoxyisoflavan] was identified as being significantly associated with a lower risk [83]. Moreover, in 1391 Korean men and women, higher plasma concentrations of genistein were significantly associated with a lower T2D risk among women who were equol producers, which is a metabolite of isoflavone, but not in men or nonequol producers [84]. Although equol is biotransformed from daidzein by the metabolism caused by gut bacteria, some individuals cannot metabolize daidzein to equol. There is increasing evidence that the endocrine-related clinical efficacy of isoflavone may be modified by equol-producing status [84].

A recent prospective analysis conducted in the PREDIMED trial showed that over a 5-year follow-up, increased total polyphenol excretion was significantly associated with lower levels of triglycerides, glucose, and diastolic blood pressure among 573 participants at high cardiovascular risk [85]. In a small cross-sectional analysis conducted among 57 Japanese women, higher plasma levels of chlorogenic acid were significantly associated with lower levels of fasting blood glucose, HbA1c, and CRP [86]. In the nationwide representative National Health and Nutrition Examination Survey (NHANES), urinary excretion of enterolignans was inversely associated with CRP levels and metabolic syndrome components [87–89].

## 7. Metagenomics

Another layer of complexity in human nutritional studies comes from the intricate interplay between the gut microbiome and dietary intake. Obesity, insulin resistance, and T2D have been correlated with an altered gut microbiota composition. The largest metagenomics-based study to date, which includes 368 Chinese individuals, identified a moderate degree of gut bacterial dysbiosis with a decline in butyrate-producing bacteria and an increase in opportunistic bacteria among T2D patients as well as an enrichment of other microbial functions conferring sulphate reduction and oxidative stress resistance [90]. This is particularly relevant because only a limited number of bacterial species have been identified as being involved in the metabolism of polyphenols [91].

The 3-week consumption of a phenolic compounds enriched virgin olive oil, containing a mixture of olive oil and thyme phenolic compounds, decreases systemic oxidized LDL and increases bifidobacterium population and microbial metabolites of phenolic compound in faeces from hypercholesterolemic humans [92]. However, this was a small randomized controlled trial only including 12 participants. For instance, in vitro digestion of water-insoluble cocoa fractions to investigate the biotransformation of polyphenols demonstrated that bacterial fermentation of the insoluble material was associated with an increase of bifidobacteria and lactobacilli as well as butyrate production [93]. Flavanols were converted into phenolic acids by the microbiota resulting in an increasing concentration of 3-hydroxyphenylpropionic acid [93]. In a separate randomized clinical trial, these microbial changes were associated with significant reductions in plasma triacylglycerol and C-reactive proteins, suggesting the potential benefits associated with the dietary inclusion of flavanol-rich foods [94].

Undoubtedly, the interplay between gut microbiome and host and its modulation by nutrition will benefit from the integration of information from a biology-wide systems approach. Integration of gene sequence, metabolomics, and other “omics” sources will pave the way towards a better molecular understanding of the complex organisms. System-wide computational approaches will aid testing mechanistic hypothesis in silico on whole systems, such as effects of diet and modulations of metabolic diseases.

## 8. Conclusions

In the past decades, active strategies for the prevention of T2D focusing on healthy diets, and lifestyle in general, have become a priority for researchers and policymakers. Growing interest has particularly emerged on the beneficial effects of plant-based diets for the prevention of chronic diseases such as obesity and diabetes [4]. Polyphenols are highly prevalent in plant-based diets, such as the MedDiet, and especially abundant in fruits, vegetables, legumes, cocoa, coffee, and red wine [7]. In addition, extra-virgin olive oil and nuts, both key components of the MedDiet, are also polyphenol-rich foods.

Given the abundant evidence from human studies regarding the intake of polyphenols, and their food sources, on related-diabetes risk factors, this narrative review has focused on well-conducted clinical trials and prospective cohort studies with special attention paid to extra-virgin olive oil, nut, and red wine consumption but also other polyphenol-rich foods. These key components of the MedDiet were inversely associated with insulin resistance and T2D risk in observational studies [46]. The intake of specific polyphenols, especially flavan-3-ols and their food sources, has demonstrated overall beneficial effects on improving insulin resistance, chronic systemic inflammation, oxidative stress, and other cardiometabolic risk factors in trials that tested effects of acute, moderate-term, or relatively long-term (up to a year) intake of flavan-3-ols [14–16]. Moreover, findings from prospective cohort studies have suggested inverse associations between the intake of total flavonoids and specific flavonoid subclasses and the risk of T2D [13, 35–37], although some controversial results still exist.

Given the chemical structures of dietary polyphenols, multiple bioactivities are displayed by interacting with molecular pathways particularly relevant for glucose homeostasis. A preliminary track of evidence for genotype-polyphenol interaction is emerging from coffee consumption and benefits on glycemia control [74]. In addition, adherence to the MedDiet, a polyphenol-rich dietary pattern, has been shown to reduce the adverse effect of the TCF7L2 polymorphism on fasting glucose [77]. The role of polyphenol-rich foods on TCF7L2 locus is therefore likely to be relevant.

The advancement in sophisticated omics methodologies has allowed the determination of molecules involved in the nutritional genomics (genetic/epigenetic, transcriptome/epigenomics, proteome, and metabolome), metagenomics, and other environmental exposures, but mainly as markers of compliance. Therefore, the integration of gene sequencing and omics techniques will lead towards a molecular understanding of the complex organisms. System-wide computational approaches will thus contribute to the study of the effects of diet on modulation of metabolic diseases by testing the hypothetical mechanisms in silico on whole systems.

In conclusion, the intake of polyphenols may be beneficial for both insulin resistance and T2D risk.

## Figures and Tables

**Table 1:** Main food sources of polyphenols.

Polyphenol	Compound	Main food sources, excluding seasoning	Main food sources, only seasoning
Total polyphenols		Coffee, oranges, apples, grapes, olives and olive oil, red wine, cocoa powder, dark chocolate, tea, black elderberry, nuts, whole grains, legumes	Cloves, dried peppermint, star anise
Flavonoids	Flavones	Virgin olive oil, oranges, whole grain wheat-flour bread, refined-grain wheat-flour bread, whole grain wheat four, black olives	Celery seed, dried peppermint, dried, common verbena
	Flavonols	Spinach, beans, onions, shallot	Capers, saffron, dried oregano
	Flavanols	Red wine, apples, peaches, cocoa powder, nuts, dark chocolate	
	Flavanones	Grapefruit/pomelo juice, oranges, orange juice, grapefruit juice	Dried peppermint, dried oregano, fresh rosemary
	Isoflavones	Soy flour, soy paste, roasted soy bean, beans	Soy sauce
	Anthocyanins	Cherries, red wine, olives, hazelnuts, almonds, black elderberry, black chokeberry, blueberries	
Phenolic acids	Benzoic acid	Olives, virgin olive oil, red wine, walnuts, pomegranate juice, red raspberry, American cranberry	Chestnut, cloves, star anise
	Cinnamic acid	Coffee, maize oil, potatoes	Dried peppermint. Common verbena, dried rosemary
Stilbenes	Resveratrol	Grapes, red wine, nuts	
Lignans		Virgin olive oil, whole grain rye flour, bread from whole grain rye flour, flaxseed	Sesame seed oil, black sesame oil, flaxseed

**Table 2:** Prospective studies evaluating polyphenol intake on the risk of type 2 diabetes.

First author, publication year, study name, location	Sex	Follow-up (years)	Age at baseline (years) (mean)	Number of cases/participants	Exposure assessment and case ascertainment	Types of polyphenols analyzed	Relative risk (95% CI) (highest versus lowest category)	Adjustment for covariates
Knekt 2002, The FMCHES, Finland	Both	18	39.3 ± 15.8	526/9878	FFQ > 100 items/identified from Social Insurance Institution Finland	Quartiles of dietary intake of major flavonoid subclasses (total flavonoid intake 24.2 mg/d)	Q4 versus Q1 0.98 (0.78, 1.24)	Sex and age
Song 2005, WHS, United States	F	8.8	≥45 (53)	1614/38,018	131-item semiquantitative validated FFQ/self-report and confirmed with supplementary questionnaire about symptoms, American Diabetes Criteria	mg/d quintiles of dietary intake of total or individual flavonols and flavones and flavonoid-rich foods	Q5 versus Q1 (median intake mg/d: 47.2 versus 8.85) of total flavonoids 0.92 (0.78, 1.09)	Age, BMI, energy, total fat, smoking, exercise, alcohol use, history of hypertension, high cholesterol, family history of diabetes, fiber intake, glycemic load, magnesium
Nettleton 2006, Iowa Women's Health Study, United States	F	18	55–69 (61)	3395/35,816	Validated 127-item FFQ/self-reported were determined by the following question: “Were you diagnosed for the first time by a doctor as having sugar diabetes?”	mg/d quintiles of flavonoid and flavonoid sources	Q5 versus Q1 (median intake mg/d: 680.4 versus 90.4) of total flavonoids 0.97 (0.86, 1.10)	Age, energy, education level, BMI, waist:hip ratio, activity level, smoking status, multivitamin use, and hormone therapy
Kataja-Tuomola 2011, ATBC, Finland	M	10.2	50–69 (57.5)	660/25,505	Validated FFQ 275 food items/self-reported or medical diagnosis and Social Insurance Institution Finland	Quintiles of flavonols and flavones	Nonsignificant associations for kaempferol, luteolin, myricetin, quercetin	Age, supplementation, BMI, cigarettes smoked daily, smoking years, blood pressure, total cholesterol, high-density lipoprotein cholesterol, leisure-time physical activity, and daily intake of alcohol and energy
Wedick 2012, NHS, NHSII, HPFS, United States	F (NHS)	24	30–55 (50)	6878/70,359	131-item semiquantitative validated FFQ/self-report and confirmed with supplementary questionnaire about symptoms, the National Diabetes Group criteria	mg/d quintiles of dietary intake of major flavonoid subclasses	Q5 versus Q1 (median intake mg/d: 718.1 versus 105.2) of total flavonoids 0.85 (0.79, 0.92)	Age, BMI, smoking status, alcohol intake, multivitamin use, physical activity, family history of diabetes, postmenopausal status and hormone use, ethnicity, total energy, intakes of red meat, fish, whole grains, coffee, high-calorie sodas, and trans fat
	F (NHSII)	16	25–42 (36)	3084/89,201	Same as above	mg/d quintiles of dietary intake of major flavonoid subclasses	Q5 versus Q1 (median intake mg/d: 770.3 versus 112.1) of total flavonoids 0.99 (0.89, 1.11)	Same as above plus oral contraceptive use
	M (HPFS)	20	40–75 (53)	2649/41,334	Same as above	mg/d quintiles of dietary intake of major flavonoid subclasses	Q5 versus Q1 (median intake mg/d: 624.3 versus 112.5) of total flavonoids 0.92 (0.81, 0.94)	Same as above except postmenopausal status and hormone use and oral contraceptive use
Zamora-Ros 2013, Epic-InterAct, 8 European countries	Both	3.99 million person-years of follow-up	52.4 (9.1)	12,403/16,154	Country-specific FFQ/self-report and linkage to primary and secondary care registers, hospital and mortality data	mg/d quintiles of dietary flavonoids, types of flavonoids and lignans intake	Q5 versus Q1 (median intake mg/d: 817.5 versus 126.8) of total flavonoids 0.90 (0.77, 1.04)	Age, sex, and total energy intake, educational level, physical activity, smoking status, BMI, alcohol intake, intakes of red meat, processed meat, sugar-sweetened soft drinks, and coffee, intakes of fiber, vitamin C, and magnesium
Jacques 2013, Framingham Offspring Cohort, United States	Both	11.9	54.2 (53.8, 54.5)	308/2915	Validated FFQ/fasting glucose concentrations and/or a medical and medication use history obtained by a physician at each study examination	6 flavonoid classes and total flavonoids	HR per 2.5-fold difference in flavonoid intake (cumulative mean flavonoid intake) 0.89 (0.75, 1.05)	Sex, age, cardiovascular disease, current smoker (y/n), BMI, and cumulative mean energy intake, vegetable and fruit intake
Zamora-Ros 2014, EPIC-InterAct, United States	Both	3.99 million person-years of follow-up	52.4 (9.1)	12,403/16,154	Country-specific FFQ/self-report and linkage to primary and secondary care registers, hospital and mortality data	mg/d quintiles of dietary flavanol and flavonol intake	Q5 versus Q1 of sum of flavanols and flavonols (median intake in mg/d: 713.6 versus 97.6). Inverse associations between all flavan-3-ol monomers, proanthocyanidin dimers and trimers (Q5 versus Q1 0.81 (0.71, 0.92) and 0.91 (0.80, 1.04), resp.)	Age, sex, and total energy intake, educational level, physical activity, smoking status, BMI, alcohol intake, intakes of red meat, processed meat, sugar-sweetened soft drinks, and coffee, intakes of fiber, vitamin C, and magnesium
Tresserra-Rimbau 2016, PREDIMED, Spain	Both	5.51	55–80	314/3430	Validated 137-item FFQ/fasting plasma glucose ≥7 mmol/L or 2 h plasma glucose ≥11.1 mmol/L after a 75 g oral glucose load, confirmed by a second test using the same criteria, the American Diabetes Association criteria	Total polyphenols, flavonoids, stilbenes, lignans	T3 versus T1 (mean intake 1002 versus 600) of total polyphenols 0.72 (0.52, 0.99)	Age, sex, recruitment center, intervention group. Smoking, BMI, physical activity, dyslipidemia, hypertension, education level, total energy intake, alcohol intake, adherence to the Mediterranean diet, and fasting glucose
Ding 2016, NHS, NHS2, HPFS, United States	F (NHS)	8	30–55 (50)	3671/63,115	131-item semiquantitative validated FFQ/self-report and confirmed with supplementary questionnaire about symptoms, the National Diabetes Group criteria	mg/d quintiles of isoflavone consumption	Q5 versus Q1 (median intake mg/d: 2.78 versus 0.17) of isoflavones 0.97 (0.88, 1.07)	Age, race, family history of T2D, baseline disease status, BMI, physical activity, overall dietary pattern (alternate Healthy Eating Index score, in quintiles), total energy intake and smoking status and menopausal status, postmenopausal hormone use
	F (NHSII)	8	25–42 (36)	3920/79,061	Same as above	mg/d quintiles of isoflavone consumption	Q5 versus Q1 (median intake mg/d: 5.73 versus 0.17) of isoflavones 0.85 (0.76, 0.95)	Same as above
	M (HPFS)	8	40–75 (53)	742/21,281	Same as above	mg/d quintiles of isoflavone consumption	Q5 versus Q1 (median intake mg/d: 5.09 versus 0.31) of isoflavones 0.80 (0.62, 1.02)	Same as above except postmenopausal status and hormone use

ATBC, *α*-Tocopherol, *β*-Carotene Cancer Prevention Study; WHS, Women's Health Study; FFQ, food frequency questionnaire; NHS, Nurses' Health Study; HPFS, Health Professionals Follow-up Study; PREDIMED, Prevención con Dieta Mediterránea; EPIC, The European Prospective Investigation into Cancer and Nutrition; FMCHES, Finnish Mobile Clinic Health Examination Survey.

## Abbreviations

CRP: C-reactive protein
HbA1c: Hemoglobin A1c
HOMA-IR: Homeostasis model assessment of insulin resistance
QUICK: Quantitative insulin sensitivity index
T2D: Type 2 diabetes.
